# Vascular endothelial growth factor: an angiogenic factor reflecting airway inflammation in healthy smokers and in patients with bronchitis type of chronic obstructive pulmonary disease?

**DOI:** 10.1186/1465-9921-8-53

**Published:** 2007-07-15

**Authors:** Nikoletta Rovina, Andreas Papapetropoulos, Androniki Kollintza, Makrina Michailidou, Davina CM Simoes, Charis Roussos, Christina Gratziou

**Affiliations:** 1 Asthma and Allergy Center, Pulmonary and Critical Care Department, Evgenidion Hospital, Medical School, University of Athens, Greece; 2 "G. P. Livanos" and "M. Simos" Laboratories, Department of Critical Care and Pulmonary Services, Evangelismos Hospital, University of Athens, Greece

## Abstract

**Background:**

Patients with bronchitis type of chronic obstructive pulmonary disease (COPD) have raised vascular endothelial growth factor (VEGF) levels in induced sputum. This has been associated with the pathogenesis of COPD through apoptotic and oxidative stress mechanisms. Since, chronic airway inflammation is an important pathological feature of COPD mainly initiated by cigarette smoking, aim of this study was to assess smoking as a potential cause of raised airway VEGF levels in bronchitis type COPD and to test the association between VEGF levels in induced sputum and airway inflammation in these patients.

**Methods:**

14 current smokers with bronchitis type COPD, 17 asymptomatic current smokers with normal spirometry and 16 non-smokers were included in the study. VEGF, IL-8, and TNF-α levels in induced sputum were measured and the correlations between these markers, as well as between VEGF levels and pulmonary function were assessed.

**Results:**

The median concentrations of VEGF, IL-8, and TNF-α were significantly higher in induced sputum of COPD patients (1,070 pg/ml, 5.6 ng/ml and 50 pg/ml, respectively) compared to nonsmokers (260 pg/ml, 0.73 ng/ml, and 15.4 pg/ml, respectively, p < 0.05) and asymptomatic smokers (421 pg/ml, 1.27 ng/ml, p < 0.05, and 18.6 pg/ml, p > 0.05, respectively). Significant correlations were found between VEGF levels and pack years (r = 0.56, p = 0.046), IL-8 (r = 0.64, p = 0.026) and TNF-α (r = 0.62, p = 0.031) levels both in asymptomatic and COPD smokers (r = 0.66, p = 0.027, r = 0.67, p = 0.023, and r = 0.82, p = 0.002, respectively). No correlation was found between VEGF levels in sputum and pulmonary function parameters.

**Conclusion:**

VEGF levels are raised in the airways of both asymptomatic and COPD smokers. The close correlation observed between VEGF levels in the airways and markers of airway inflammation in healthy smokers and in smokers with bronchitis type of COPD is suggestive of VEGF as a marker reflecting the inflammatory process that occurs in smoking subjects without alveolar destruction.

## Background

Chronic obstructive pulmonary disease (COPD) is characterized by slowly, progressive and largely irreversible airflow limitation due to chronic bronchitis, emphysema, or both [1]. Long-term cigarette smoking is the most important risk factor that may initiate the disease, as a result of inflammatory cells into the lung (leading to chronic airway inflammation), imbalance between proteolytic and anti-proteolytic activity, oxidative stress and apoptosis [2].

The appearance of chronic progressive airflow limitation in part reflects lung remodeling [3]. Vascular endothelial growth factor (VEGF) is the most potent directly acting regulator of angiogenesis [4,5], and a trophic factor that is required for the survival of endothelial cells, inducing endothelial cell proliferation [6], while its withdrawal leads to endothelial cell apoptosis [7,8]. VEGF's expression is often excessive in chronic inflammation and fibrosis, and it has been implicated in the pathogenesis of emphysema through apoptotic and oxidative stress mechanisms [9-11]. Cigarette smoking may upregulate VEGF, as suggested by an acute increase of VEGF plasma levels during smoking [12]. Although there is increasing evidence of the implication of VEGF in the pathogenesis of COPD its role at different stages of the disease seems to be controversial; it is suggested that it has a detrimental function in the bronchi and a protective role in the alveoli [13].

Although chronic inflammation is considered the hall-mark of COPD, little data exists about the role of VEGF in the inflammatory process involved in the pathogenesis of the disease, especially in current smokers. On this basis, the aim of this study was not only to assess VEGF levels in induced sputum of healthy smokers and of smokers with bronchitis type of COPD, but to further assess smoking as a potential cause of raised airway VEGF levels in bronchitis type of COPD, and to test the association between VEGF levels in induced sputum and airway inflammation in these subjects.

## Methods

### Subjects

Fourteen smokers with COPD, seventeen asymptomatic healthy current smokers and sixteen non-smoking controls were included in the study. All asymptomatic smokers were lifelong smokers (> 15 pack-years), with no history of lung disease, no chronic respiratory symptoms, and normal spirometry. All COPD patients were current smokers (> 15 pack-years), with chronic cough and sputum production over at least 3 months for 2 successive years, and irreversible airflow limitation (reversibility < 10% predicted forced expiratory volume in 1 second (FEV_1_) after 200 μg of inhaled salbutamol). All patients satisfied the ERS criteria [14] for COPD and were selected according to the Global Initiative for Chronic Obstructive Lung Disease (GOLD) [15] to fulfill the criteria for GOLD stages I and II COPD (FEV_1_/FVC ≤ 0.7, and FEV_1 _≥ 0.8 and 0.8 ≤ FEV_1 _≤ 0.7, respectively) and to have no evidence of emphysema, based on high-resolution computed tomographic scans of the lungs and the diffusing capacity of lung for carbon monoxide (DL*CO*).

All participants met the following criteria: no use of inhaled or oral corticosteroids in the previous 6 months, no atopy (negative skin prick tests for 10 common aeroallergens), and no respiratory tract infection 1 month prior to the study. None of the COPD patients was ever hospitalized due to an exacerbation of COPD. None of the healthy non-smokers and smokers was receiving either long acting bronchodilators or leukotriene modifiers. Nine out of fourteen COPD patients were under treatment with inhaled tiotropium and inhaled short acting beta agonists per need, five were receiving inhaled short acting beta agonists or ipratropium per need, and none was receiving long acting bronchodilators or leukotriene modifiers. Before each measurement subjects were asked not to use long or short-acting β_2 _agonists and/or ipratropium at least 12 hours prior to the tests, and tiotropium 48 hours prior to the tests.

All subjects gave informed consent for participation in the study, which was approved by the Hospital ethics committee.

### Measurements

All subjects visited the hospital on 3 separate days, at least 2 days apart. Lung function tests (flow-volume curves, reversibility test, diffusing lung capacity for carbon monoxide (DL*CO*), measurement of arterial blood gases, skin prick tests, and sputum induction were performed.

### Lung function

Lung function (FEV_1, _FEV_1_/FVC) was measured with a dry wedge spirometer (Masterscreen, Jaeger, Hoechberg, Germany) according to standardized guidelines [16], by the same technician using the same spirometer. Reversibility test was performed 20 minutes after inhalation of 200 μg salbutamol via a metered dose inhaler. The diffusing lung capacity for carbon monoxide (DL*CO*) was measured by the single breath method at least twice (Masterscreen, Jaeger, Hoechberg, Germany).

### Sputum induction and processing

Sputum was induced by inhalation of hypertonic saline aerosol and processed as described previously [17]. Briefly, 15 minutes after salbutamol inhalation (200 μg), normal saline 0.9% and then hypertonic saline (3%, 4% and 5%) nebulized by an ultrasonic nebulizer (ULTRA-NEB 2000, DeVilbiss Heathcare INC, Somerset, USA) was inhaled for each concentration over a period of 7 minutes. Subjects were encouraged to cough deeply after the 7-minute intervals. All subjects produced an adequate aliquot of sputum which was processed within 2 hours after termination of the induction. The sputum was diluted threefold with phosphate buffer solution containing dithiothreitol (final concentration: 1 mmol/L) and centrifuged at 790 g for 4 minutes (4°C), and the pellet was resuspended. Slides were made by using cytospin (Cytospin 3, Shandon, INC, Pittsburgh, USA). Two sputum cytospin slides were stained with May-Grünwald-Giemsa for differential cell counts. Counting of 400 non-squamous cells took place in a blinded way by one technician. Sputum samples containing > 20% of squamous cells were excluded from analysis as indication of poor cytospin quality. The supernatant was stored at -80°C for subsequent assay for IL-8, TNF-α, and VEGF concentrations, which were measured using an enzyme-linked immunosorbent assay kit (ELISA) (R & D Systems, Minneapolis, Minnesota, USA).

### Statistical analysis

Data were expressed in mean (± SD) or median values. Inflammatory markers and VEGF were expressed in median values and inter-quartile range. Differences between subjects' groups were initially assessed by Kruskal-Wallis test, and if significant, the Mann-Whitney rank test was then assessed. Correlations between inflammatory cells and mediators in sputum, smoking characteristics or lung function parameters were calculated with Spearman's rank correlation test. Statistical analysis was not influenced by values at the lower limits of detection since the non-parametric tests used were based on ranks of values.

A p value of less than 0.05 was considered significant.

## Results

Clinical characteristics of subjects participated in the study are in Table 1. All subjects were matched for age, and smokers had similar mean values for smoking pack years, arterial oxygen tension, DLCO (% pred), FRC (% pred), RV (% pred), and TLC (% pred). However, FEV_1 _and FEV_1_/FVC were significantly lower (p < 0.001, Table 1) in COPD smokers (mean ± SD, 68 ± 11%), compared to healthy non smokers (106 ± 12%) and asymptomatic smokers (101 ± 9%).

**Table 1:** Clinical characteristics and lung function parameters of study subjects

**No**	**Healthy non-smokers 16**	**Asymptomatic smokers 17**	**COPD smokers n = 14**
Age (years)	46 ± 11	47 ± 8	54 ± 9
Smoking (pack-years)	0	34 ± 6	45 ± 17
FEV_1 _(% pred)	106 ± 12*	101 ± 9*	68 ± 11
FVC (% pred)	107 ± 10*	108 ± 11*	85 ± 15
FEV_1_/FVC (%)	85 ± 6*	79 ± 6*	64 ± 5
FEF_25–75 _(% pred)	94 ± 31*	79 ± 20*	32 ± 10
RV (% pred)	99 ± 13	92 ± 9	85 ± 25
TLC (% pred)	97 ± 8	96 ± 8	89 ± 12
FRC (% pred)	98 ± 16	95 ± 14	95 ± 15
DLCO (% pred)	91 ± 9	85 ± 10	89 ± 11
PaO_2 _(mm Hg)	-	96 ± 11	81 ± 14

Values are expressed as mean ± SD.

COPD: chronic obstructive pulmonary disease; FEV_1_: forced expiratory volume in one second; FVC: forced vital capacity; FEF_25–75_: forced expiratory flow 25–75; RV: residual volume; TLC: total lung capacity; FRC: forced residual capacity; DLCO: diffusing lung capacity for carbon monoxide; PaO_2_: partial pressure of oxygen, arterial.

*p < 0.05, asymptomatic smokers and healthy non-smokers vs COPD smokers

### Sputum

The median (inter-quartile range) total number of cells in COPD smokers) was higher (though not significantly, p > 0.05) compared to asymptomatic smokers and significantly higher (p < 0.05) compared to non smokers (Table 2). Smokers with COPD had higher percentage of sputum neutrophils compared to asymptomatic smokers (p < 0.05), and non smokers (p < 0.05). In contrast, the percentage of sputum macrophages was significantly lower in COPD smokers compared to asymptomatic smokers (p < 0.05), and non smokers (p < 0.05) (Table 2).

**Table 2:** Sputum inflammation in asymptomatic and COPD smokers as compared to healthy non-smokers

**No**	**Healthy non-smokers 16**	**Asymptomatic smokers 17**	**COPD smokers 14**
**Total no of cells × 10^4^**	28.5 (23–53)*	41 (27–115)	56 (25–133)
**Cell concentration ml × 10^4^**	14 (11–18)*	22.5 (13–57)	28 (12–66)
**Macrophages, %**	56 (50–68)*	48 (42–62)*	22 (14–43)
**Neutrophils, %**	37 (27–47)*	48.5 (34–55)*	70 (47–82)
**Lymphocytes, %**	3.4 (1.6–5.6)	2.5 (0.6–5.5)	2.5 (0.21–6.4)
**Eosinophils, %**	0.46 (0.32–0.6)	0.35 (0.1–0.9)	1.25 (0.58–3.2)

Values are median (inter-quartile range).

*p < 0.05 vs COPD smokers; ¶p < 0.05 vs asymptomatic smokers

**Figure 1:**
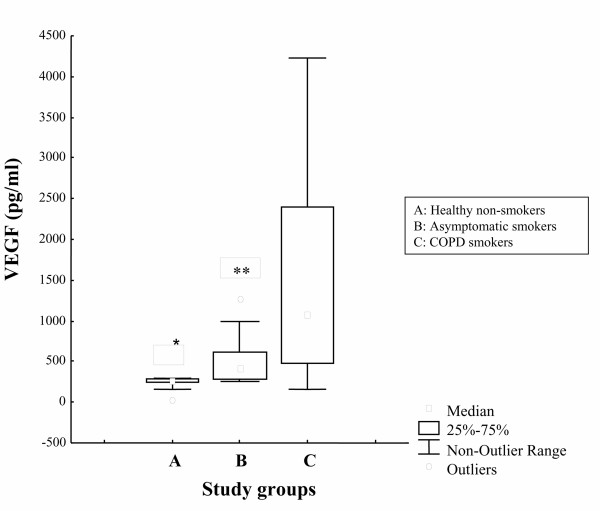
Levels of VEGF (pg/ml), expressed as median values (inter-quartile range) in induced sputum of healthy non-smokers (A), asymptomatic smokers (B) and COPD smokers (C). *p < 0.05, for healthy non-smokers vs COPD smokers; **p < 0.05, for asymptomatic vs COPD smokers.

The concentration of VEGF in induced sputum was significantly higher in COPD smokers than in asymptomatic smokers (p = 0.024) and healthy non-smokers (p = 0.002) (Figure 1).

**Figure 2:**
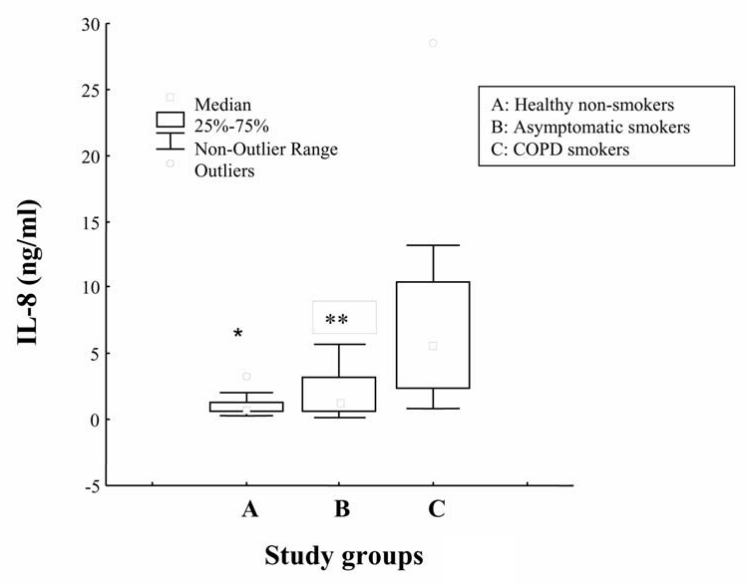
Levels of IL-8 (ng/ml), expressed as median values (inter-quartile range) in induced sputum of healthy non-smokers (A), asymptomatic smokers (B) and COPD smokers (C). *p < 0.05, for healthy non-smokers vs COPD smokers; **p < 0.05, for asymptomatic vs COPD smokers.

**Figure 3:**
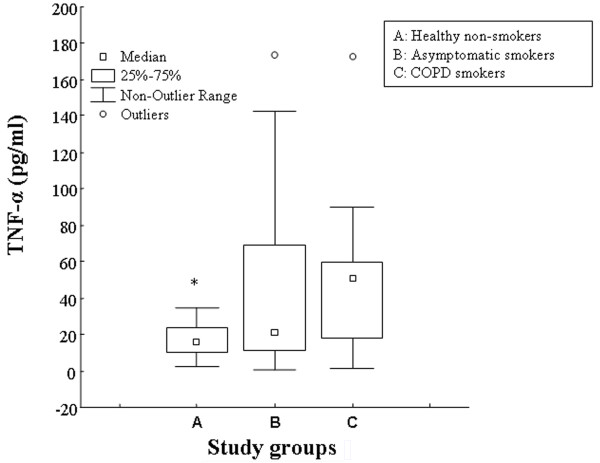
Levels of TNF-α (pg/ml), expressed as median values (inter-quartile range) in induced sputum of healthy non-smokers (A), asymptomatic smokers (B) and COPD smokers (C). *p < 0.05, for healthy non-smokers vs COPD smokers.

Levels of IL-8 and TNF-α in induced sputum of COPD smokers [5.6 ng/ml (2.3–10), and 50 pg/ml [17–75], respectively] were higher compared to asymptomatic smokers [1.27 ng/ml (0.72–3.2), p = 0.021, and 18.6 pg/ml [10–35], p = 0.322, respectively] and non smoking subjects [0.73 ng/ml (0.6–1.4), p = 0.000, and 15.4 pg/ml [9–25], p = 0.014, respectively] (Figures 2, 3).

**Figure 4:**
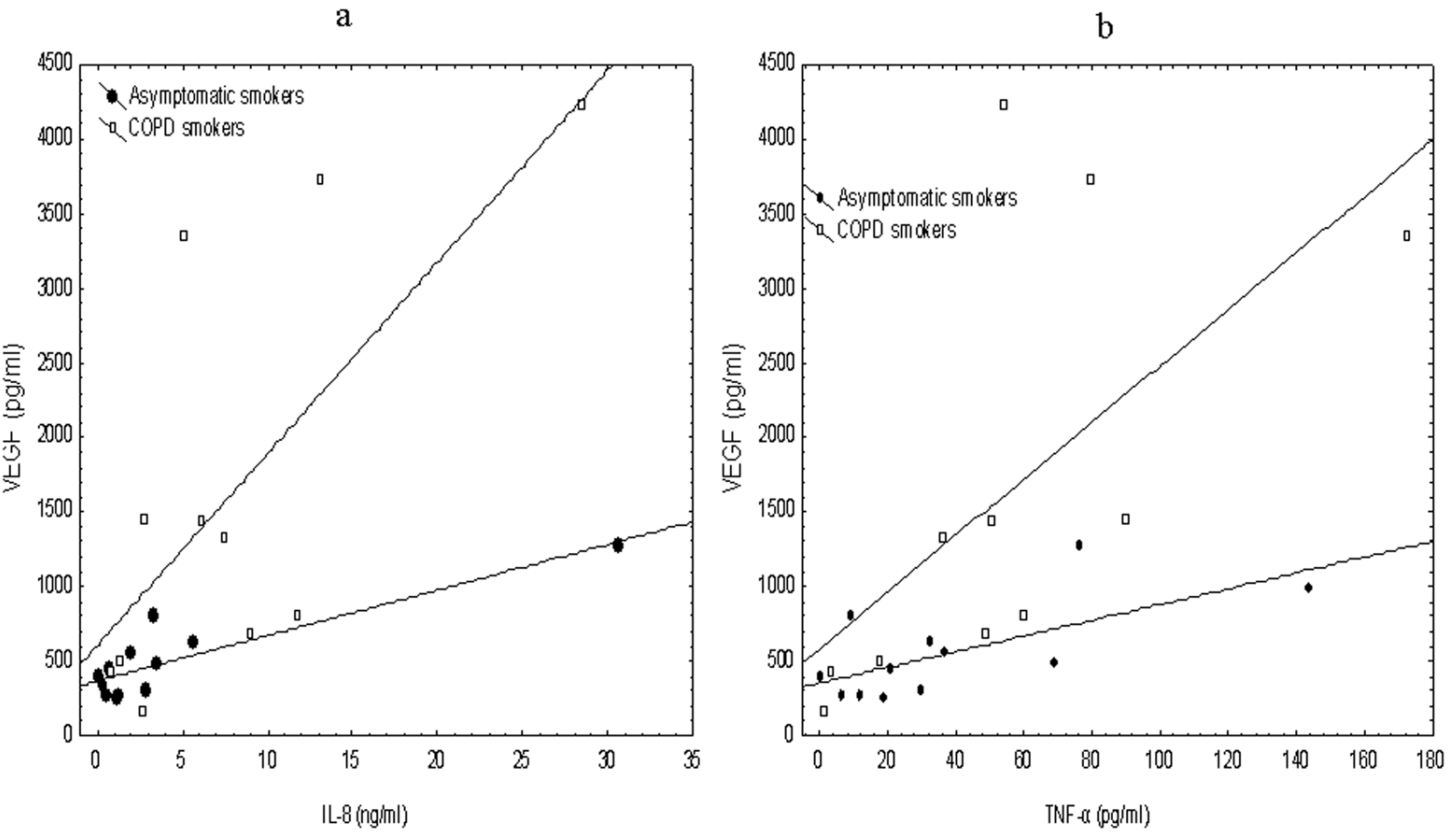
Spearman's rank correlation: VEGF and IL-8 levels in induced sputum of asymptomatic (r = 0.636, p = 0.026) and COPD smokers (r = 0.673 and p = 0.023). **4b**. Spearman's rank correlation: VEGF and TNF-α levels in induced sputum of asymptomatic (r = 0.622, p = 0.031) and COPD smokers (r = 0.818 and p = 0.002).

The VEGF levels in induced sputum in both groups of smokers (asymptomatic and COPD smokers) were significantly correlated with smoking pack years, with IL-8 and TNF-α levels (Table 3, Figures 4a and 4b).

**Table 3:** Spearman's rank correlations between VEGF levels in induced sputum and smoking pack-years, airway obstruction, and airway inflammation in asymptomatic and COPD smokers

	**Asymptomatic smokers n = 17**		**COPD smokers n = 14**	
	r	p-value	r	p-value

***VEGF levels in induced sputum***				
Smoking pack-years	0.56	0.046	0.66	0.027
FEV_1_, % pred	-0.20	NS	-0.13	NS
DL*CO*, % pred	0.524	NS	0.00	NS
Macrophages in sputum, %	-0.261	NS	0.046	NS
Neutrophils in sputum, %	0.246	NS	0.430	NS
IL-8 in sputum, ng/ml	0.636	0.026	0.673	0.023
TNF-α in sputum, pg/ml	0.622	0.031	0.818	0.002

FEV_1_: forced expiratory volume in one second; DLCO: diffusing lung capacity for carbon monoxide; VEGF: vascular endothelial growth factor; IL-8: interleukin-8; TNF-α: tumor necrosis factor alpha.

No significant correlation was found between VEGF levels in sputum and pulmonary function parameters in COPD patients.

## Discussion

The main finding of this study is that cigarette smoking is an important determinant of vascular endothelial growth factor (VEGF) upregulation in the airways, as assessed by VEGF's levels in induced sputum, since it correlated significantly with pack years but not with other clinical and functional parameters. Furthermore, this VEGF upregulation correlated positively with increased levels of inflammatory mediators, such as interleukin-8 (IL-8) and tumor necrosis factor-α (TNF-α) in sputum not only in mild COPD smokers, but also in asymptomatic smokers.

It has been long recognized that exposure to cigarette smoke causes cellular oxidative stress and release of inflammatory mediators in the airways of healthy subjects and that these effects can be both acute and chronic [18-20]. Compared to healthy non-smokers the degree of airway inflammation is higher in COPD patients irrespective of whether these patients are current or ex-smokers [21-25]. In bronchitis type of COPD airway inflammation is characterized by an influx of inflammatory cells, predominantly neutrophils, macrophages, and CD8+ T lymhocytes, into the airway walls [26], and is associated with structural alterations including an increase in the amount of smooth muscle and connective tissue in the airway wall [27]. Furthermore, previous studies have indicated that pulmonary arteries in patients with chronic bronchitis have increased adventitial infiltration of activated T lymphocytes [28,29]. Therefore, active airway inflammation might affect pulmonary vascular remodeling in chronic bronchitis. In turn, angiogenesis of bronchial vasculature, has been shown to increase the recruitment of inflammatory cells and the exudation of mediators in the airways [4,27], resulting in a vicious cycle of intracellular signalling between inflammatory and angiogenesis mediators. Vascular endothelial growth factor (VEGF) is the most potent directly acting regulator of angiogenesis [4,5] which is produced by various cell types. Macrophages, neutrophils, epithelial cells, fibroblasts, and smooth muscle cells are all important sources of VEGF in inflamed tissue [30]. Many inflammatory mediators [prostaglandin E_1 _(PGE_1_), PGE_2_, TNF-α, IL-1, IL-6, IL-8, nitric oxide, and platelet-activating factor], and pathophysiological conditions (hypoxia, pulmonary hypertension) have been shown to induce the expression of VEGF, angiogenesis, or both [30,31]. Interestingly, in our study elevated levels of VEGF were found both in asymptomatic and COPD smokers, indicating that the stimulus of chronic exposure to smoke might be the mainstream trigger for the VEGF upregulation. Cigarette smoking may upregulate the expression of VEGF in the airways, as suggested by acute increase in VEGF levels during smoking [32]. Although the increase in neutrophils by smoking may explain the increase of VEGF, it may also be the result of increased VEGF. The lung epithelia can produce VEGF which could act as a chemokine for neutrophils [33,34].

Conklin and colleagues [35] have also shown that in current smokers, nicotine and cotinine upregulate VEGF in endothelial cells, while Wright and colleagues [36] demonstrated upregulation of VEGF gene expression and its receptor (Flk-1) in pulmonary arteries of rats exposed to cigarette smoke.

Although, increased airway VEGF levels were found in both groups of smokers (healthy and COPD smokers) these were significantly higher in COPD smokers, probably due to the effects of current smoking superimposed upon the ongoing underlying inflammatory process of COPD. In the present study, higher number of inflammatory cells, percentage of neutrophils and levels of IL-8 and TNF-α were demonstrated in the induced sputum of COPD smokers compared to asymptomatic smokers, in concordance with previous observations [24,25,37,38]. The role of TNF-α and IL-8 in smoking induced airway diseases has been demonstrated in several studies [22-24]. IL-8 is a potent activator of neutrophils [39], while TNF-α is a powerful pro-inflammatory cytokine that is a key mediator of inflammation, and has an important role in fibrogenesis [40]. TNF-α activates macrophages, and epithelial and mesenchymal cells to produce various inflammatory cell chemo-attractants such as IL-8, MCP-1, and leukotriene B_4 _[41,42], and has been implicated in the smoke induced influx of macrophages and connective tissue breakdown [43]. It is suggested that the higher levels of VEGF found in COPD smokers might be the result of a cross talk between VEGF and inflammatory mediators participating in the underlying ongoing pathophysiologic procedure of the disease. The close correlation found between VEGF levels and inflammatory mediators which interfere with smoking related airway disease supports this suggestion and indicates that VEGF may be actively implicated in the pathogenesis of COPD commencing at the initial stages of the disease. However, our results cannot establish whether the increased levels of VEGF are the cause or the consequence of increased inflammatory mediators' levels found in the sputum of both groups of smokers (healthy and COPD smokers). Hypoxia, a major factor involved in the induction of VEGF gene expression does not seem to be implicated here, since the majority of our patients had normal arterial oxygen tension.

Another aspect reasoning this observation could be that VEGF and its receptor system may contribute to the maintenance of endothelial and epithelial cell viability in response to injury caused by smoking. This hypothesis is supported by the finding of Kranenburg and colleagues [44] that increased VEGF expression and their receptors (VEGFR-1, also called FLT-1, and VEGFR-2 also called KDR/Flk-1) were demonstrated in ex-smoking patients with COPD in comparison with ex-smoking healthy control subjects. Furthermore, in COPD patients, increased numbers of macrophages with increased KDR/Flk-1 and TGF-β expression were found in the bronchiolar airway epithelium [45]. Taken together, these data suggest that TGF-β-VEGF represents a molecular link between inflammatory cell infiltration at sites of smoking-induced injury contributing to airway remodeling in COPD through tissue repair mechanisms. It seems that in the lungs of COPD patients interactions between inflammatory, oxidative stress and apoptotic mechanisms most probably take place. Recenty, Kanazawa and colleagues [46] suggested that VEGF levels in induced sputum could be possibly used as a non-invasive marker of pulmonary vascular remodeling in patients with bronchitis type of COPD, indicating that VEGF may have a potential role in the pathogenesis of the vascular changes that take place in this group of patients. The results of this study support the findings of the present study, since Kanazawa and colleagues hypothesized that vascular remodeling in the pulmonary arteries of bronchitis type of COPD could be related to the inflammatory process caused by smoking and disease.

In the present study no correlation was found between airflow limitation and VEGF. A possible explanation could be that our patients, who had mild bronchitis type of COPD, were axiomatically homogeneous for disease severity. In the study of Kanazawa and colleagues [13], the COPD patients had much lower FEV_1, _and impaired DLCO compared to our subjects. Furthermore they all were ex-smokers. This is the first study examining the correlation of VEGF with pulmonary function and inflammatory mediators in current smokers with mild COPD and intact alveolar structure. If our study included COPD patients with a wider degree of airflow obstruction, a correlation between VEGF levels and functional parameters (i.e.FEV_1 _and FEV_1_/FVC) might be demonstrated.

## Conclusion

In conclusion, cigarette smoking seems to be the major determinant of vascular endothelial growth factor (VEGF) upregulation in the airways even before the occurrence of respiratory symptoms. The fact that VEGF upregulation positively correlates with the increased levels of inflammatory mediators in sputum not only in bronchitis type of COPD smokers but also in asymptomatic smokers, may indicate that VEGF plays an important signalling role linking the inflammatory milieu with changes in bronchial epithelium and endothelium quite early in smokers' airways. It is likely that, in the progression of COPD, phenomena that are the result of complex regulatory abnormalities play a central role, in which VEGF might be a key factor.

## Competing interests

All authors of this paper declare that they have no financial or other potential conflicts of interest concerning the subject of this manuscript.

## Authors' contributions

NR and MM performed all the clinical measurements of the study. NR, AP and CG provided intellectual input, writing and review of the data and paper. NK and DS analysed the sputum samples. CR reviewed the paper. All authors read and approved the final manuscript.
